# Developing a prognostic model using machine learning for disulfidptosis related lncRNA in lung adenocarcinoma

**DOI:** 10.1038/s41598-024-63949-1

**Published:** 2024-06-07

**Authors:** Yang Pan, Xuanhong Jin, Haoting Xu, Jiandong Hong, Feng Li, Taobo Luo, Jian Zeng

**Affiliations:** 1 Department of Pulmonary Surgery, Zhejiang Cancer Hospital, Hangzhou Institute of Medicine (HIM), Chinese Academy of Sciences, Hangzhou, China; 2 Postgraduate Training Base Alliance of Wenzhou Medical University (Zhejiang Cancer Hospital), Hangzhou, China; 3 Department of Medical Oncology, Sir Run Run Shaw Hospital, College of Medicine, Zhejiang University, Hangzhou, China; 4 School of Medicine, Shaoxing University, Shaoxing, China

**Keywords:** The Cancer Genome Atlas, Gene Expression Omnibus, Disulfidptosis, Lung adenocarcinoma, Machine learning, Biochemistry, Cancer, Computational biology and bioinformatics, Genetics, Biomarkers, Oncology

## Abstract

Disulfidptosis represents a novel cell death mechanism triggered by disulfide stress, with potential implications for advancements in cancer treatments. Although emerging evidence highlights the critical regulatory roles of long non-coding RNAs (lncRNAs) in the pathobiology of lung adenocarcinoma (LUAD), research into lncRNAs specifically associated with disulfidptosis in LUAD, termed disulfidptosis-related lncRNAs (DRLs), remains insufficiently explored. Using The Cancer Genome Atlas (TCGA)-LUAD dataset, we implemented ten machine learning techniques, resulting in 101 distinct model configurations. To assess the predictive accuracy of our model, we employed both the concordance index (C-index) and receiver operating characteristic (ROC) curve analyses. For a deeper understanding of the underlying biological pathways, we referred to the Kyoto Encyclopedia of Genes and Genomes (KEGG) and Gene Ontology (GO) for functional enrichment analysis. Moreover, we explored differences in the tumor microenvironment between high-risk and low-risk patient cohorts. Additionally, we thoroughly assessed the prognostic value of the DRLs signatures in predicting treatment outcomes. The Kaplan–Meier (KM) survival analysis demonstrated a significant difference in overall survival (OS) between the high-risk and low-risk cohorts (p < 0.001). The prognostic model showed robust performance, with an area under the ROC curve exceeding 0.75 at one year and maintaining a value above 0.72 in the two and three-year follow-ups. Further research identified variations in tumor mutational burden (TMB) and differential responses to immunotherapies and chemotherapies. Our validation, using three GEO datasets (GSE31210, GSE30219, and GSE50081), revealed that the C-index exceeded 0.67 for GSE31210 and GSE30219. Significant differences in disease-free survival (DFS) and OS were observed across all validation cohorts among different risk groups. The prognostic model offers potential as a molecular biomarker for LUAD prognosis.

## Introduction

Lung cancer represents over 11% of global cancer diagnoses and a significant 18.4% of cancer-induced deaths, establishing it as the most prevalent and deadliest cancer type^1^. It primarily includes small cell lung cancer (SCLC) and non-small cell lung cancer (NSCLC)^2^. In the past decade, the treatment landscape for lung adenocarcinoma (LUAD), a subtype of NSCLC, has dramatically transformed, primarily due to the development of immune checkpoint inhibitors (ICIs)^3^. These ICIs target proteins such as cytotoxic T-lymphocyte antigen 4 (CTLA-4), programmed cell death protein-1 (PD-1), and programmed death-ligand-1 (PD-L1), significantly improving survival rates for LUAD patients^4^. However, immunotherapy remains effective for only a minority of these patients^5^, highlighting the urgent need to identify predictive biomarkers for accurate prognostic assessments.

In tumor immunology, disulfidptosis, a sophisticated metabolism-associated regulated cell death, has been identified. This process involves a series of disulfide bond formations between proteins, both within and outside cells, leading to changes in protein structure and function, ultimately resulting in cell death^6^. New therapeutic strategies targeting the glucose transporter (GLUT) family of glucose transporters have shown potential in inducing disulfidptosis and inhibiting cancer cell growth^7^. Yet, the complex relationships among disulfidptosis, LUAD prognosis, the tumor microenvironment (TME), and treatment responses present rich opportunities for further investigation.

Moreover, research emphasizes the crucial role of long non-coding RNAs (lncRNAs) in cancer management^8^. LncRNAs regulate essential biological processes in various cancers, including lung cancer, affecting cell proliferation, invasion, and metastasis^9^. Recent studies have linked lncRNAs to resistance against tyrosine kinase inhibitors (TKIs) in NSCLC, especially in cases involving linear plasticity^10^. In lung cancer, lncRNAs significantly influence the tumor microenvironment, with specific lncRNAs like SNHG5 inhibiting LUAD progression by modulating the epithelial-mesenchymal transition^11,12^. Despite their established role in pathogenesis, research on disulfidptosis-related lncRNAs (DRLs) in lung cancer prognosis and treatment sensitivity remains groundbreaking.

Using next-generation sequencing and advanced bioinformatics, our research marks a pioneering step in molecular oncology. We are deeply examining the complex molecular interactions and signaling pathways mediated by lncRNAs to enhance risk stratification models, aiming to improve prognostic accuracy for LUAD patients. By introducing this innovative lncRNA signature model derived from LUAD datasets, we are forging a new path in precision medicine to optimize treatments for LUAD patients.

## Methods

### Collection and handling of public access data

**Figure 1:**
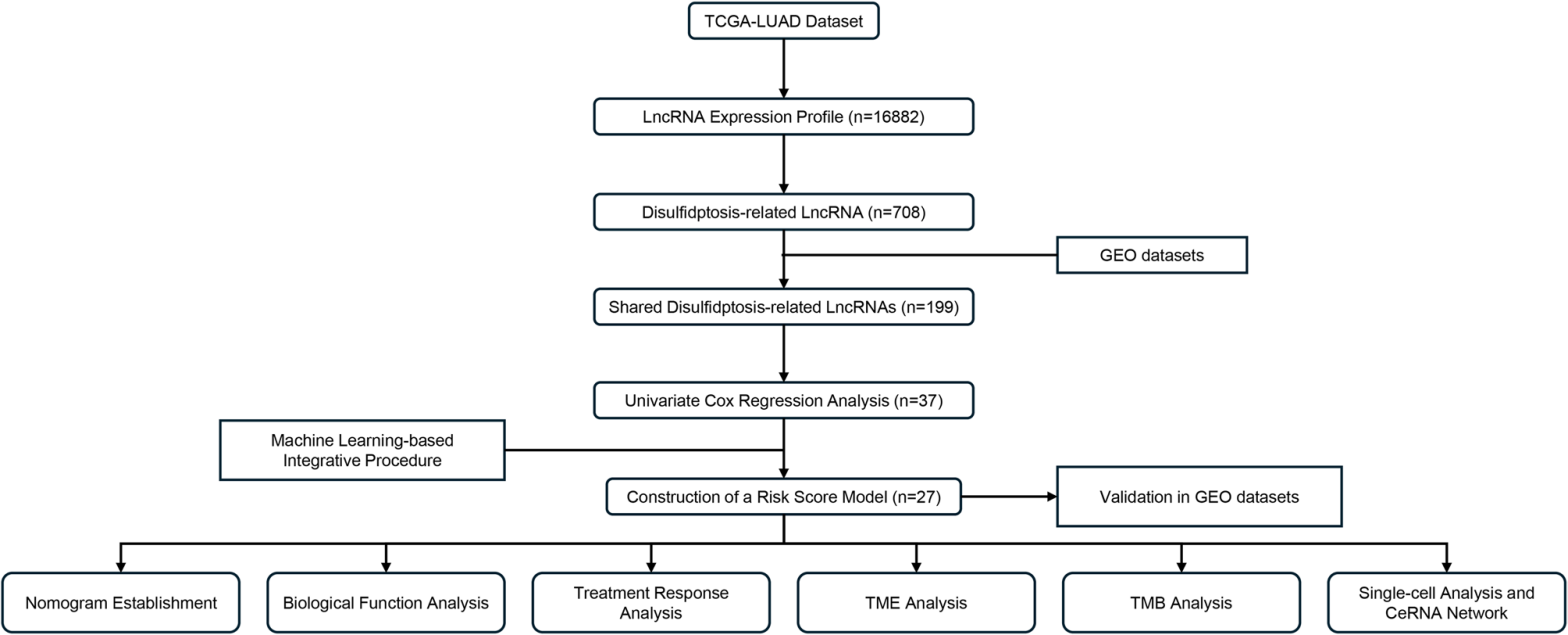
Overview of the study's analytic process.

After excluding patients with incomplete clinical records, we analyzed data from 916 LUAD patients derived from The Cancer Genome Atlas (TCGA) datasets [https://portal.gdc.cancer.gov/] and Gene Expression Omnibus (GEO) [https://www.ncbi.nlm.nih.gov/geo/] datasets. The transcripts per million (TPM)-normalized expression dataset for TCGA-LUAD was retrieved using the “easyTCGA” package. To enhance the interpretability of expression values, we applied a logarithmic transformation with a base of 2. Data from the GEO were obtained from the Affymetrix® GPL570 platform, employing the Human Genome U133 Plus 2.0 Array for gene expression analysis. The raw data from Affymetrix® were processed with the robust multiarray averaging method using the "Affy" R package. We corrected for batch effects using the ComBat algorithm and merged three GEO datasets to form a comprehensive Meta-GEO cohort. Expression values for each gene were standardized as z-scores across all patient cohorts. A flowchart depicting the study methodology is presented in Fig. 1, and detailed data from the three GEO datasets are provided in Supplementary Table 1.

### Prognostic model construction

In our comprehensive literature review, we identified 24 genes associated with disulfide-triggered cell death^6^. Using the "limma" package, we examined the relationship between 24 disulfidptosis-related genes (DRGs) and lncRNAs, identifying significant lncRNAs associated with disulfidptosis (p-value < 0.010, |Correlation|> 0.3). These lncRNAs, combined with data from the GEO database, formed the basis of our model. To enhance model accuracy and robustness, we implemented a novel strategy starting with a univariate regression analysis of lncRNAs from TCGA-LUAD to identify potential prognostic lncRNAs. Firstly, we initiated a univariate regression analysis on the sequentially identified lncRNAs from TCGA-LUAD, pinpointing potential prognostic-linked lncRNAs. We then designated TCGA-LUAD as our training set and the three GEO datasets as validation. We engaged ten machine learning methods—Ridge Regression, Least Absolute Shrinkage and Selection Operator (Lasso) Regression, Stepwise Cox Regression, CoxBoost, Random Survival Forests (RSF), Elastic Net (Enet), plsRcox, Supervised Principal Components (SuperPC), survival-Support Vector Machines (SVM), and Gradient Boosting Machine (GBM). Each model was chosen for its unique strengths and ability to complement the analysis in various ways:

(1) Ridge Regression: Known for its ability to handle multicollinearity, Ridge regression applies L2 regularization to prevent overfitting, making it suitable for our dataset with a high dimensionality of genomic features.

(2) Lasso Regression: Lasso employs L1 regularization which not only helps in reducing overfitting but also in feature selection by shrinking some coefficients to zero. This is particularly useful in identifying the most relevant DRLs influencing LUAD progression.

(3) Stepwise Cox Regression: This method applies both forward and backward selection techniques to identify significant variables in survival analysis, ideal for modeling the impact of DRLs on patient survival.

(4) CoxBoost: CoxBoost adjusts for high-dimensional data by incrementally fitting the Cox model, enhancing its predictive accuracy in our setting where numerous potential predictors are involved.

(5) RSF: An extension of the random forest that handles censored data, RSF is adept at dealing with complex interactions and non-linear relationships, crucial for understanding intricate DRL interactions.

(6) Enet: Combining the properties of both Ridge and Lasso, Elastic Net is effective when dealing with datasets where numerous features are correlated, providing a balanced approach to regularization and variable selection.

(7) plsRcox: This model is useful for reducing dimensionality while maintaining the relationship with the survival outcome, helpful in elucidating the impact of DRLs on LUAD.

(8) SuperPC: SuperPC is employed to enhance predictive accuracy by focusing on principal components most associated with survival outcomes, refining our analysis of DRLs' prognostic power.

(9) survival-SVM: This model adapts the traditional SVM for survival analysis, which is beneficial for handling non-linear patterns in the data, providing a robust classification of risk groups.

(10) GBM: GBM builds an ensemble of decision trees sequentially, improving the model iteratively, which is key in accurately classifying patients into risk categories based on their DRL profiles.

These methods were amalgamated in 101 unique combinations for variable selection and model formulation. Finally, we evaluated the efficacy of our model on the training and validation datasets employing the concordance index (C-index), subsequently calculating an average score. The optimal selection was the Enet model with α = 0.2. Leveraging the survminer package, we determined the risk score threshold from the surv_cutpoint for the LUAD-TCGA dataset, subsequently segmenting both TCGA-LUAD and the three GEO datasets into low-risk and high-risk groups.

### Analysis of survival outcomes and development of a prognostic nomogram

Utilizing the “survminer” package in R, we conducted Kaplan–Meier (KM) curve analyses on TCGA-LUAD and three GEO datasets, probing the disparities in LUAD patients' overall survival (OS) and disease-free survival (DFS) between the high-risk and low-risk cohorts. To evaluate the potential of the risk score as a standalone prognostic marker, we utilized univariate and multivariate cox analyses. With the “timeROC” package, we generated receiver operating characteristic (ROC) curves for one, two, and three-year durations for the risk score taken from TCGA-LUAD and the three GEO datasets, subsequently computing the area under the ROC curve (AUC). We delved into the relationship between risk score and patient attributes, including TNM staging, gender, smoking history, and mutations in the EGFR/KRAS/ALK genes. Additionally, we devised a nomogram that amalgamates age, gender, staging, and risk score to maximize predictive accuracy. The clinical relevance of this nomogram was then gauged using calibration and decision curve analysis (DCA).

### Analysis of tumor mutational burden (TMB)

The TMB was sourced from the TCGA-LUAD database. TMB quantification was carried out utilizing the “tmb” function from the “maftools” package. For visualization and in-depth analysis, a waterfall plot was employed to delineate the top 10 genes exhibiting the highest TMB in patients diagnosed with TCGA-LUAD. Moreover, a survival curve analysis was conducted to discern the disparities in OS among patient groups categorized based on distinct TMB levels.

### Functional enrichment analysis

We conducted a differential expression analysis with criteria set at |logFC|> 1 and p-value < 0.050 to identify significant differences between low-risk and high-risk cohorts. The differentially expressed genes (DEGs) identified through this analysis were then subjected to functional enrichment analysis. Specifically, we used Gene Ontology (GO)^13–15^ enrichment to identify relevant biological processes, molecular functions, and cellular components. We utilized the GO database for Gene Set Enrichment Analysis (GSEA). Additionally, we performed pathway enrichment analysis using the Kyoto Encyclopedia of Genes and Genomes (KEGG) database [https://www.genome.jp/kegg/] to uncover key signaling pathways associated with the DEGs.

### Tumor microenvironment analysis

We utilized tools like CIBERSORT, MCP-counter, EPIC, TIMER, xCell, and quanTIseq to probe differences in the TME between high-risk and low-risk cohorts. The analysis was streamlined using the R package “IOBR”, and the results were visualized as heatmaps via the “pheatmap” package. Further, we displayed CIBERSORT data using box plots, highlighting variations in immune cell infiltration between high-risk and low-risk cohorts. Drawing from our prior research^16^, we used a select set of 28 genes, recognized for representing immune cells, and carried out single sample genome enrichment analysis (ssGSEA). In our final steps, we utilized the “ESTIMATE” algorithm to quantify the components of immune and stromal cells in the TME of each LUAD patient.

### Immunotherapy and chemotherapy sensitivity analysis

We tapped into the Tumor Immune Dysfunction and Exclusion (TIDE) scores, noted for its predictive accuracy in immune checkpoint blockade (ICB) treatment outcomes^17^, alongside the Immune Prognostic Scores (IPS), a marker of immunogenicity. Data was sourced from the TIDE [http://tide.dfci.harvard.edu/] and The Cancer Immunome Atlas (TCIA) [https://tcia.at/home]. We juxtaposed the IPS and TIDE scores across our patient cohorts. Building on a study from Cancer Medicine^18^, we also identified genes linked to tumor-infiltrating lymphocytes (TLS), pivotal for immunotherapy success. To probe the relationship between risk scores and ICB treatment outcomes, we leveraged a compilation of 79 immune checkpoint genes (ICGs) drawn from prior research^19^. Individual samples received unique scores based on both TLS and ICG gene sets, and scores from the high-risk and low-risk cohorts were then compared. Exploring the signature's potential for targeted drug therapy in LUAD, we gauged the half maximal inhibitory concentration (IC50) of pertinent chemotherapeutic agents using the “pRRophetic” R package. We then employed Wilcoxon's test to discern IC50 variations between the risk groups.

### Single-cell analysis and ceRNA network prediction

We carried out a differential gene analysis using GTEx and TCGA-LUAD on the Xena Functional Genomics Explorer (https://xenabrowser.net/). We subsequently performed a single-cell analysis utilizing the TISCH database (http://tisch.comp-genomics.org/home/) and conducted a comprehensive ceRNA analysis. We began by using DIANA-LncBase v3 (www.microrna.gr) to identify miRNAs targeted by LINC00857. We then used these miRNAs to target the corresponding mRNAs in miRTarBase (http://miRTarBase.cuhk.edu.cn/).

### Statistical analysis

We employed the R 4.3.1 software for statistical analysis. To assess the relationship between lncRNAs and DRGs, we applied the pearson correlation method. To construct prognostic models, we first conducted univariate cox regression to assess the prognostic significance of each lncRNA individually. LncRNAs with a p-value less than 0.05 in the univariate analysis were subsequently included in the machine learning methods. The model's predictive accuracy was gauged using DCA curves, C-index, ROC analysis, and the calibration curves, while KM analysis shed light on the survival dynamics of distinct groups. Our data underwent scrutiny using a mix of statistical tests such as the Chi-square, Wilcoxon, and Log-rank tests. The choice of test was based on data characteristics, distribution, and the number of groups in comparison. For all tests, statistical significance was denoted by a p-value below 0.05.

## Results

### Identification of prognostically relevant DRLs and construction of prognostic models

**Figure 2:**
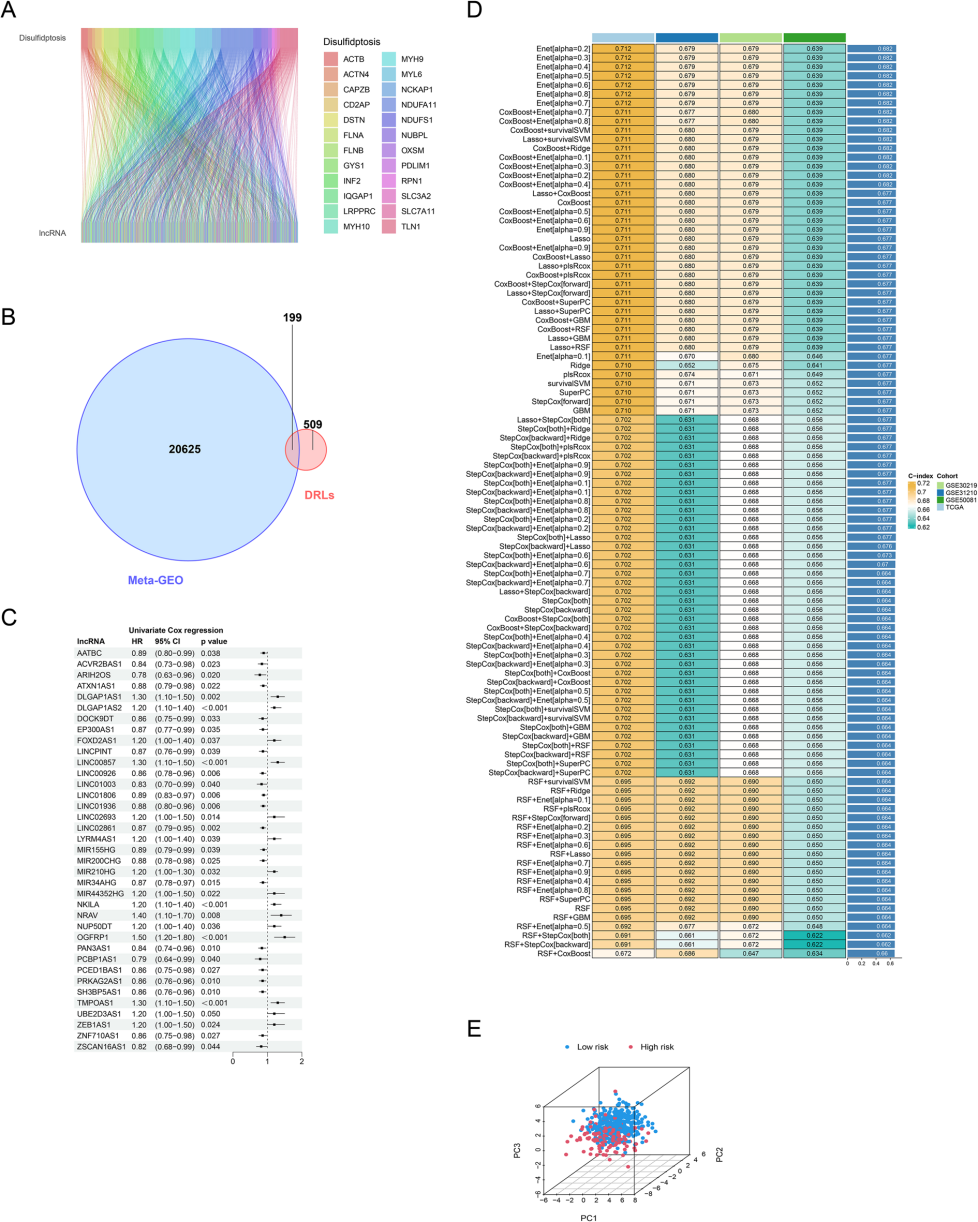
Construction of prognostic model composed of 27 DRLs. (**A**) Sankey diagram illustrating the relationship between DRGs and associated lncRNAs. (**B**) The intersection of DRLs sourced from the TCGA database and GEO database. (**C**) 27 lncRNAs after univariate Cox regression. (**D**) 101 prediction models evaluated, with C-index calculated for each across all validation datasets. (**E**) Principal Component Analysis of the low-risk and high-risk cohorts based on 27 DRLs.

In our investigation of the LUAD landscape, we analyzed 16,882 lncRNAs derived from the TCGA-LUAD database. This comprehensive evaluation led to the identification of 708 DRLs, which demonstrate significant interactions with DRGs, as depicted in a sankey diagram (Fig. 2A). Through further analysis incorporating data from three GEO databases, we narrowed these DRLs down to 199 lncRNAs consistently present across datasets, suggesting a pivotal role in LUAD pathogenesis (Fig. 2B). Our prognostic assessment using univariate cox regression analysis revealed 37 lncRNAs with significant implications for LUAD patient outcomes (Fig. 2C). Leveraging these lncRNAs, we constructed a predictive model employing an ensemble of machine learning techniques, with the ensemble model (Supplementary Table 2) achieving a notably high C-index of 0.677[95% confidence interval (CI) 0.63 to 0.73], suggesting robust predictive performance (Fig. 2D). This model's effectiveness was further validated through a risk stratification system, categorizing patients into high and low-risk groups based on their lncRNA expression profiles. This stratification was substantiated by principal component analysis (PCA), which confirmed the distinct separation between the risk groups, underscoring the potential of our model in clinical risk assessment (Fig. 2E).

### Efficacy of the LUAD prognostic model

**Figure 3:**
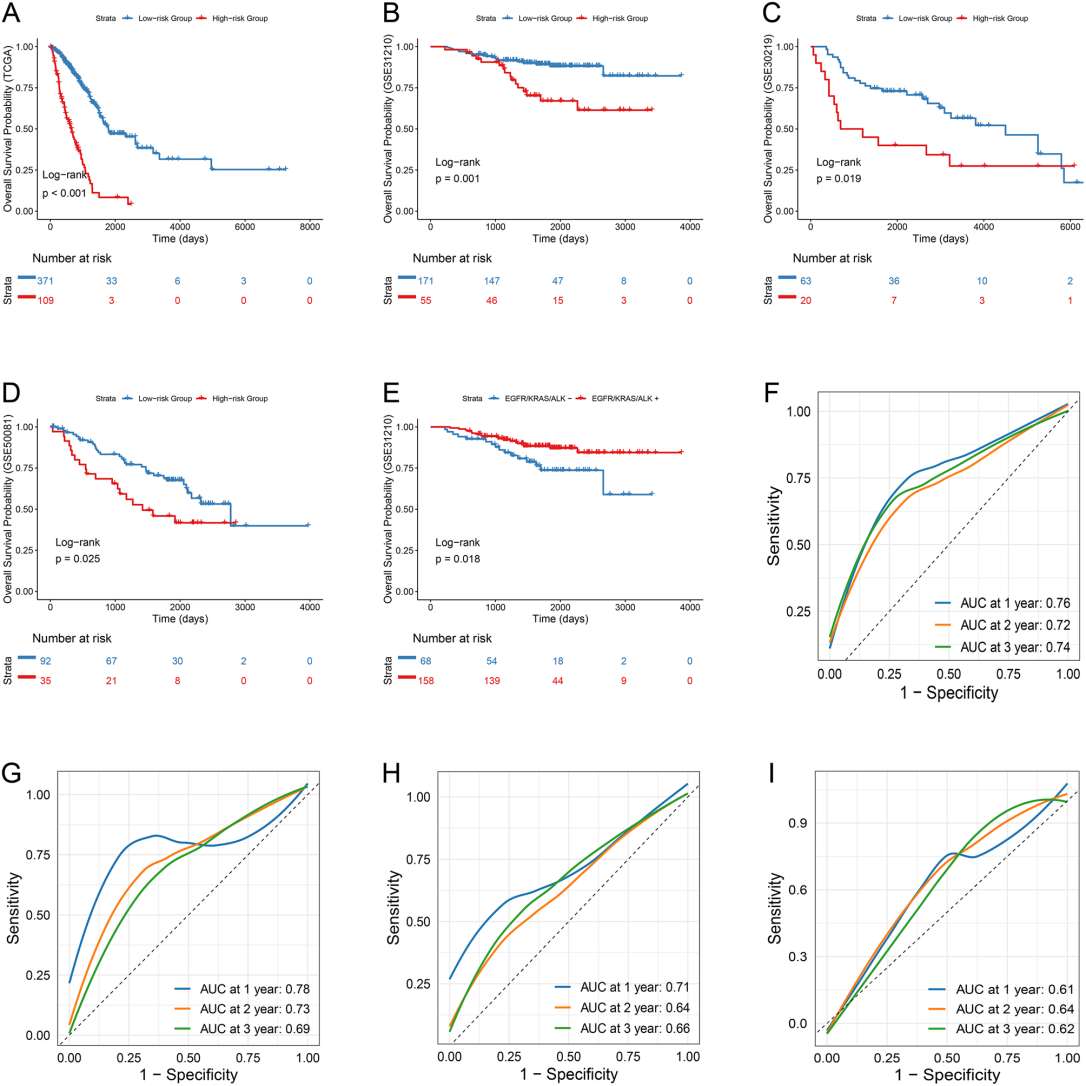
Efficacy of the DRLs Survival Prognostic Risk Model. Kaplan–Meier (K–M) analysis for high-risk and low-risk groups are exhibited in (**A**) TCGA-LUAD, (**B**) GSE31210, (**C**) GSE30219 and (D)GSE50081. (**E**) Kaplan–Meier (KM) survival curves for mutant and non-mutant groups. Analysis of 1-, 2-, and 3-year ROC curves for (**F**) TCGA-LUAD, (**G**) GSE30219, (**H**) GSE50081, and (**I**) GSE31210.

Our survival analysis using the TCGA-LUAD dataset revealed a significant distinction in OS between the high- and low-risk groups identified through our model (p < 0.001, log-rank test) (Fig. 3A). This finding was consistently replicated across three independent GEO datasets, demonstrating significant differences in both OS (GSE31210, p = 0.001; GSE30219, p = 0.019; GSE50081, p = 0.025) (Fig. 3B–D) and DFS (GSE31210, p < 0.001; GSE30219, p = 0.009; GSE50081, p = 0.023) (Supplementary Fig. S1A–C). The predictive power of the risk score was superior to that of traditional prognostic factors such as age, gender, and staging, as evidenced by the C-index comparison (Supplementary Fig. S1D). The risk score also emerged as an independent prognostic indicator in our univariate and multivariate cox analyses (p < 0.001) (Supplementary Table 3). Multicollinearity within the model was assessed using the variance inflation factor, which was below 10 for all variables (Supplementary Table 4). The AUC analysis further validated the robustness of our model, with one-year, two-year, and three-year AUCs of 0.76, 0.72, and 0.74, respectively, in the TCGA-LUAD dataset (Fig. 3F). The external validation using GEO datasets underscored the model's accuracy, particularly notable in GSE30219, GSE50081 and GSE31210 for the evaluated intervals (Fig. 3G,I).

Further analysis showed gender-specific differences in risk scores across various pathological stages. In early stages (I and II), men exhibited significantly higher risk scores compared to women (Stage I: p = 0.015; Stage II: p = 0.006; Wilcoxon test) (Supplementary Fig. S2A,B). However, these differences were not observed in later stages (III/IV) (p = 0.900, Wilcoxon test) (Supplementary Fig. S2C), suggesting stage-specific risk dynamics. In addition, our study uncovered notable disparities in risk scores among patients with mutations in EGFR, ALK, and KRAS genes in the GSE31210 dataset (p < 0.001, Kruskal–Wallis test) (Supplementary Fig. S2D). Patients harboring these mutations also exhibited better OS compared to those without (p = 0.018, log-rank test) (Fig. 3E), highlighting the potential prognostic relevance of genetic profiles in LUAD. The impact of smoking, a known risk factor for LUAD, was evident as significant differences in risk scores between smokers and non-smokers were observed in analyses of the GSE30210 and GSE50081 datasets (GSE31210, p = 0.003; GSE50081, p = 0.027; Wilcoxon test) (Supplementary Fig. S2E,F).

### Construction and validation of nomogram

**Figure 4:**
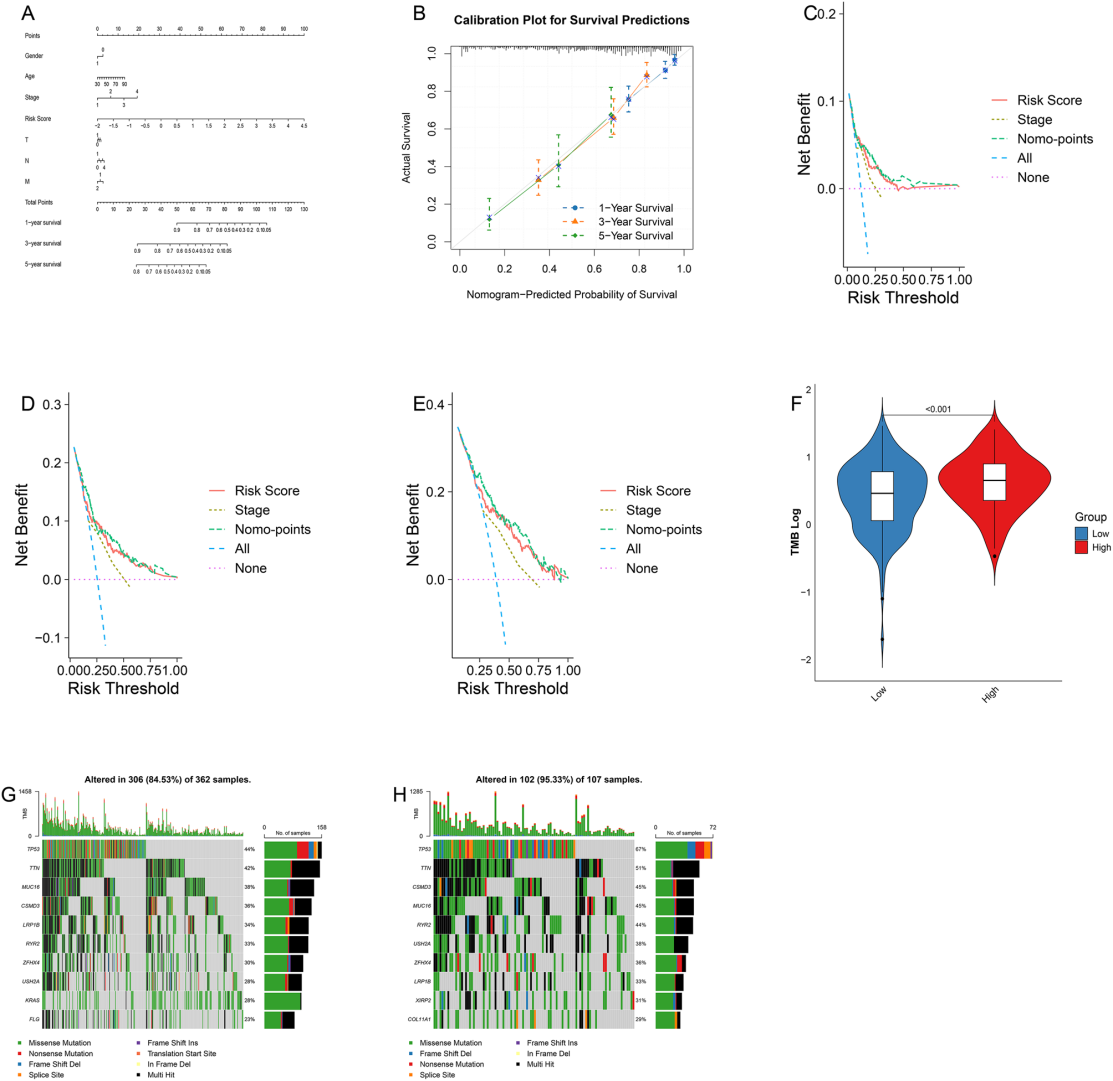
Development of a Nomogram for Risk Prediction & Analysis of Mutation Patterns in Both Risk Groups. (**A**) Nomogram that combines model and clinicopathological factors. (**B**) Calibration curves in 1-, 3-, and 5-year for the nomogram. (**C**–**E**) The decision curves analysis (DCA) of the nomogram and clinical characteristics in 1-, 3-, and 5-year. (**F**) TMB levels between the high-risk and low-risk groups. (**G**) Gene mutation waterfall chart of the low-risk group. (**H**) Gene mutation waterfall chart of the high-risk group.

To enhance our model's utility in clinical decision-making, we developed a nomogram that incorporates the identified risk scores alongside essential clinical parameters—age, gender, and TNM staging. This integration aims to provide a more comprehensive tool for predicting the prognosis of LUAD patients (Fig. 4A). We rigorously validated the nomogram's predictive accuracy using calibration curves, which compare the predicted survival probabilities against the observed outcomes. The results demonstrated a high degree of concordance, indicating that our nomogram accurately reflects patient survival rates (Fig. 4B). Further assessment through DCA (Fig. 4C-E) confirmed that the nomogram provides substantial clinical benefit. Notably, the analysis showed that the nomogram significantly outperforms the predictive capabilities of the risk score alone, particularly in terms of net benefit across a wide range of threshold probabilities.

### Discrepancy in TMB levels between LUAD's high-risk and low-risk cohorts

A marked difference in TMB was discerned between the high- and low-risk cohorts (p < 0.001 by wilcoxon test) (Fig. 4F). The waterfall plot delineates the mutational landscape of the ten most prevalent genes across both risk strata. In the low-risk cohort, approximately 84.53% of specimens exhibited gene mutations (Fig. 4G), whereas in the high-risk stratum, mutations were observed in roughly 95.33% of specimens (Fig. 4H). Predominant mutations within the high-risk category included TP53, TTN, and CSMD3.

### Enrichment analysis of the pathway

**Figure 5:**
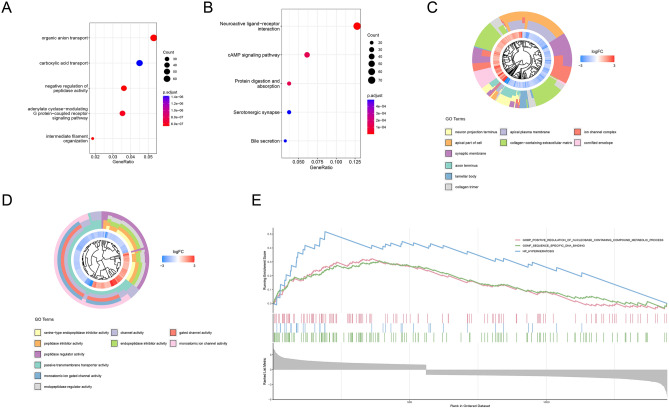
Biological function analysis of the DRLs risk score model. The top 5 significant terms of (**A**) GO function enrichment and (**B**) KEGG function enrichment. (**C**,**D**) System clustering dendrogram of cellular components. (**E**) Gene set enrichment analysis.

The differential expression analysis revealed a total of 1474 DEGs between the low-risk and high-risk cohorts. Among these, 568 genes were upregulated and 906 genes were downregulated. The volcano plot (Supplementary Fig. S2G) illustrates the distribution of these DEGs. These results indicate that specific genes are significantly associated with risk stratification in our study cohort. In the GO analysis (Fig. 5A,D), DEGs showed predominant enrichment in terms of molecular functions such as organic anion transport, carboxylic acid transport. Regarding cellular components, the main enrichment was observed in the apical plasma membrane (Fig. 5C). Figure 5E demonstrates the GSEA results, highlighting significant enrichment of specific gene sets related to metabolic processes, DNA binding, and hyperkeratosis. The KEGG result highlighted a significant enrichment of DEGs in neuroactive ligand-receptor interaction and the cAMP signaling pathway (Fig. 5B).

### Immuno-infiltration analysis

**Figure 6:**
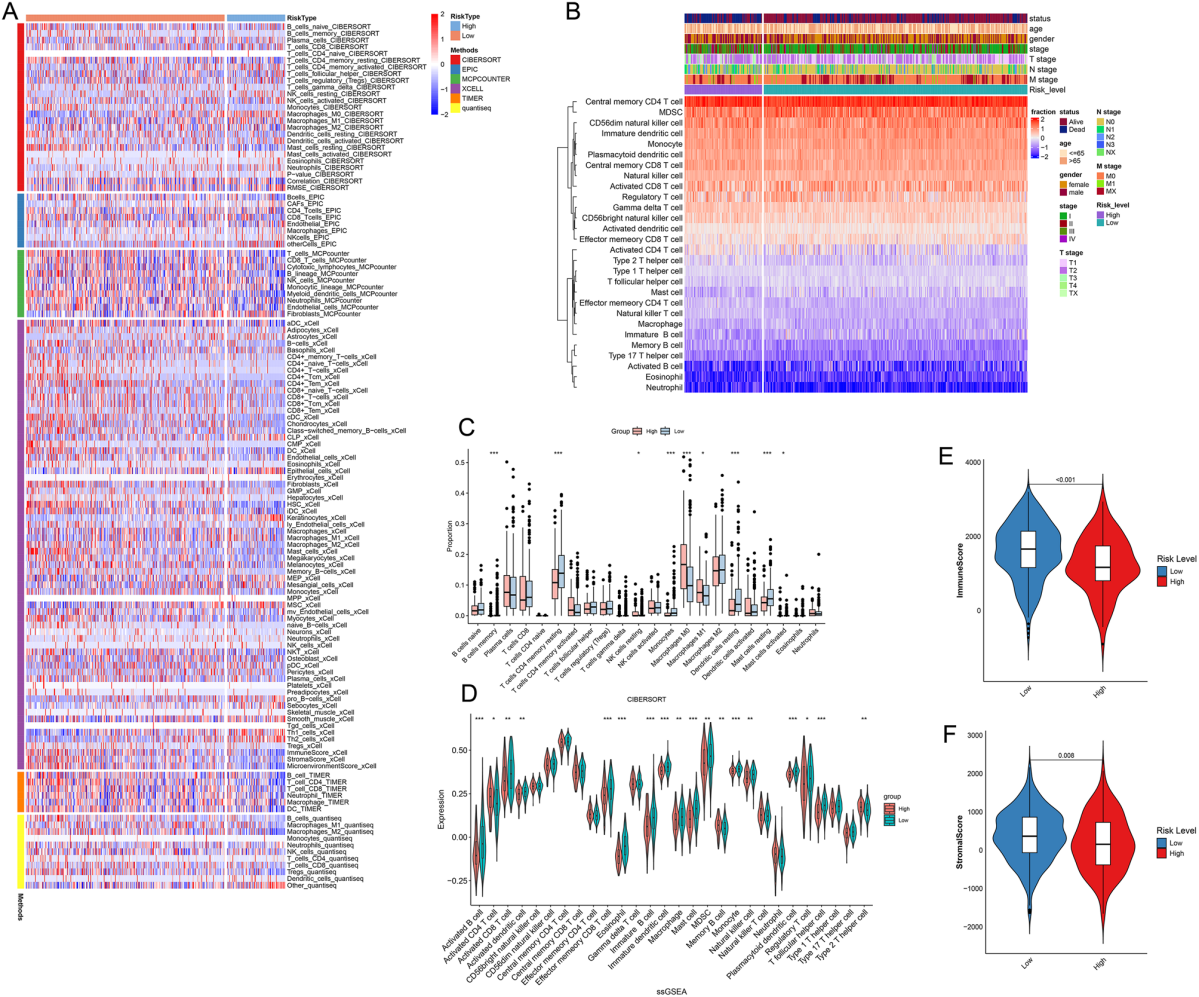
The tumor microenvironment between high-risk and low-risk groups based on DRLs. (**A**) Comparing the levels of immune cell infiltration for different immune cell types in the CIBERSORT, EPIC, MCP-counter, xCell, TIMER and quanTIseq algorithm for low-risk and high-risk groups. (**B**) Immune infiltration of different lung adenocarcinoma patient characteristics. Box plot of the difference in immune cell infiltration between the high-risk and low-risk score groups based on (**C**) CIBERSORT and (**D**) ssGSEA. *p-value < 0.05, **p-value < 0.01, ***p-value < 0.001, ns = no significance. (**E**) Immune score, and (**F**)stromal score were lower in the high-risk group than in the low-risk group.

To validate the precision of our results, we employed seven techniques: CIBERSORT, EPIC, MCP-counter, xCell, TIMER, quanTIseq, and ssGSEA, to assess immune cell penetration in both high-risk and low-risk categories (Fig. 6A). With the ssGSEA data, we explored the connection between TME and several characteristics of lung adenocarcinoma patients, such as age, gender, and disease stage (Fig. 6B). We then visualized this data with box plots for both CIBERSORT and ssGSEA (Fig. 6C,D). These plots showed that the infiltration levels of B cells memory, T cells CD4 memory resting, and Monocyte was notably lower in the high-risk group compared to the low-risk group. With the help of the “ESTIMATE” algorithm, we evaluated the stromal (Fig. 6F), immune (Fig. 6E), and ESTIMATE scores (Supplementary Fig. S3A) across the different risk groups. This allowed us to gauge tumor purity. Our study suggests that the high-risk group has reduced stromal, ESTIMATE, and immune scores. Conversely, the score of tumor purity in the low-risk group is less than that in the high-risk group (Supplementary Fig. S3B).

### Investigating the relationship between the predictive model and sensitivity to immunotherapy

**Figure 7:**
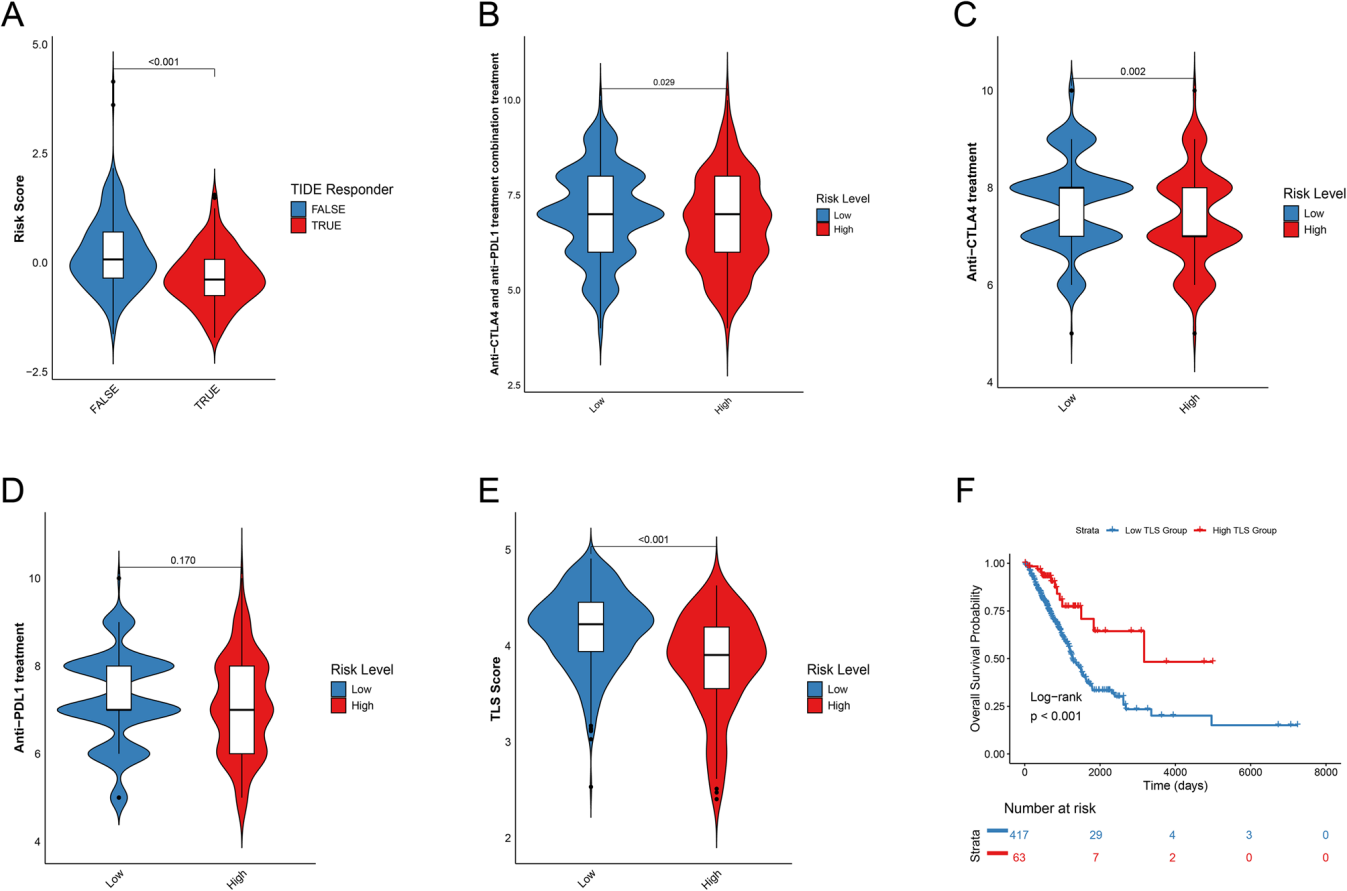
Immunotherapeutic sensitivity between high-risk and low-risk groups based on DRLs. (**A**) Differences in risk scores between the TIDE responsive and nonresponsive groups. (**B**–**D**) Sensitivity of high- and low-risk groups to combination therapy, anti-CTLA4, and anti-PDL1 by different IPS scores. (**E**) Differences in tumor tertiary lymphoid structure (TLS) scores in high-risk and low-risk groups in TCGA-LUAD. (**F**) KM analysis of high-TLS and low-TLS groups.

We calculated the TIDE score and forecasted the immunotherapy response in both groups of the high risk and low risk (Fig. 7A). Based on results from both datasets, patients in low-risk group seem more inclined to show a positive reaction to immunotherapy. Additionally, IPS for the combination of anti-CTLA4 and anti-PDL1 treatment, as well as for anti-CTLA4 alone, was consistently higher in the low-risk group (Fig. 7B,C). However, the analysis of anti-PDL1 treatment alone (P = 0.170) did not reach statistical significance (Fig. 7D). This suggests that low-risk patients may respond better to anti-CTLA4 and/or anti-PDL1 immunotherapy. Recently, research has found a link between tumor TLS and outcomes in several tumor types. In line with these discoveries, our review of TCGA-LUAD dataset showed that LUAD patients with high TLS scores had more favorable outcomes than those with low scores (Fig. 7F). We also noticed that the TLS score was higher in the low-risk group compared to the high-risk group (Fig. 7E).

### Chemotherapy response in high-risk vs. low-risk groups

**Figure 8:**
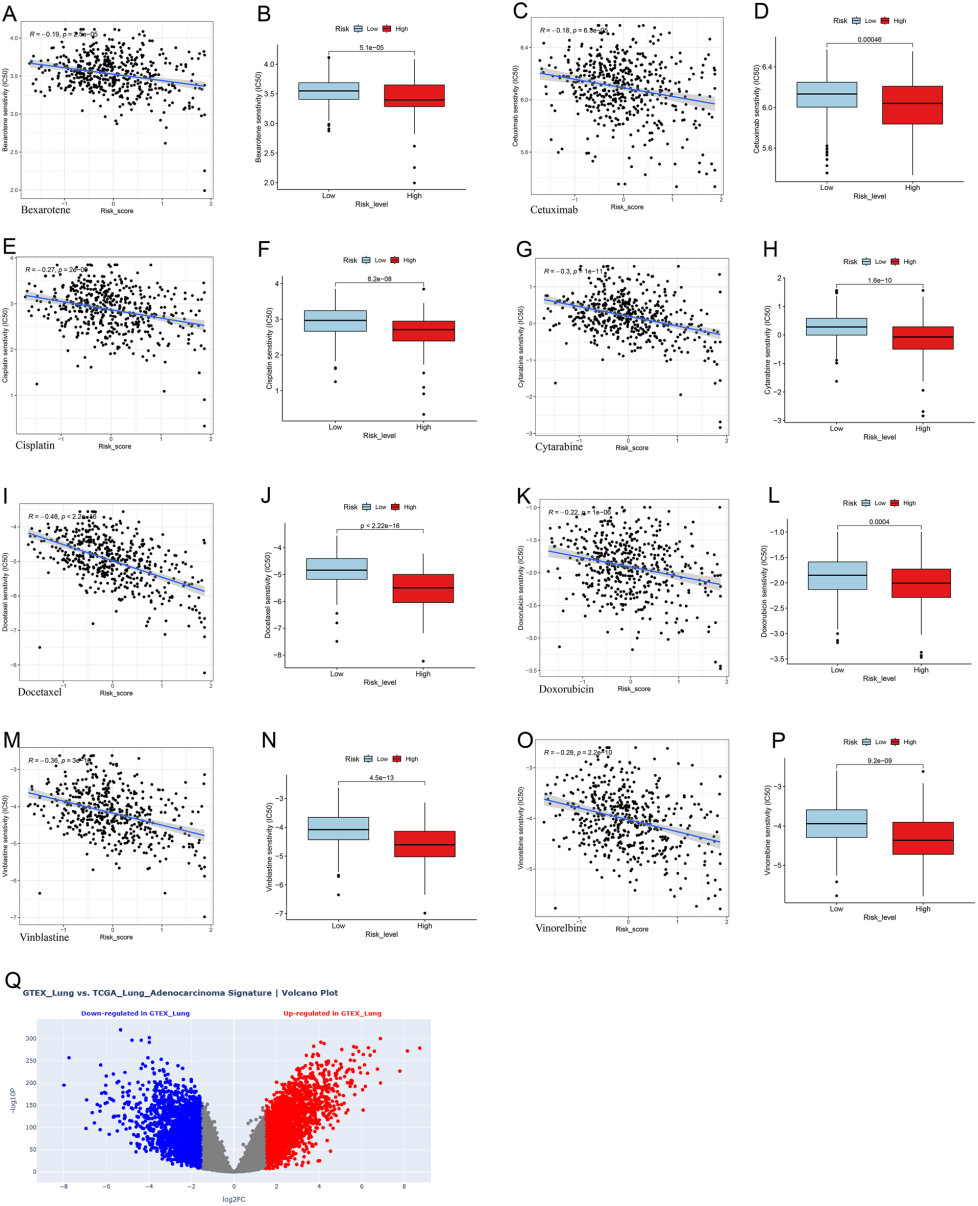
Immunotherapy sensitivity analysis and in-depth study of LINC00857. (**A**–**P**) Differences in drug sensitivity between high-risk and low-risk groups. (**Q**) Volcano plot for GTEX_Lung vs. TCGA_Lung_ Adenocarcinoma.

In our assessment of the relationship between risk scores and sensitivity to chemotherapy, we measured the IC50 for some widely used chemotherapeutic medicine. Our findings showed that the high-risk group was more sensitive to drugs like Cisplatin, Vinblastine, Cytarabine, Vinorelbine, Bexarotene, Cetuximab, Docetaxel, and Doxorubicin than the low-risk group (Fig. 8A–P).

### Single-cell expression levels of LINC01003

Through differential gene analysis of tumor tissues and normal tissues, 13,995 DEGs (|logFC|> 1.5, p-value < 0.050) (Fig. 8Q, Supplementary Fig. S3C) were identificated. By cross-referencing with the 27 lncRNAs that form our prognostic model, we pinpointed LINC01003. Supplementary Fig. S4A presents a heatmap demonstrating the expression levels of LINC01003 across different NSCLC datasets and cell types. The results indicate that LINC01003 is differentially expressed, with notable high expression in monocytes/macrophages and endothelial cells across several datasets, suggesting its potential involvement in these cell types within the NSCLC tumor microenvironment. Supplementary Figure S4B further illustrates the expression profile of LINC01003 in different cell populations from the GSE143423 dataset. The violin plot shows significant expression of LINC01003 in malignant cells, compared to other cell types, indicating its potential role in tumor progression.

### CeRNA network of LINC00857

To decipher the LINC00857 related regulatory mechanisms, we constructed a lncRNA-miRNA-mRNA network (Supplementary Fig. S4C). This network illustrates the intricate interactions between LINC00857 and various miRNAs and mRNAs. In this network, LINC00857 acts as a central regulatory hub, potentially influencing gene expression by sequestering multiple miRNAs, such as hsa-miR-4709-5p, hsa-miR-760, and hsa-miR-340-5p. These miRNAs, in turn, are connected to a wide array of target genes, including YWHAZ, BCL2L2, PTEN, and MYC, which are critical in cellular processes such as cell cycle regulation, apoptosis, and signal transduction.

## Discussion

Disulfidptosis, a newly recognized form of cell death, is closely linked to tumor development and progression^20^. A thorough understanding of its regulatory pathways could pave the way for precision therapies in oncology^21^. Lung cancer remains a major concern globally, being the most frequently diagnosed cancer and the leading cause of cancer-related deaths^22^. Annually, about 2 million new lung cancer cases are reported, with LUAD constituting a significant proportion of these cases^23^. Precise global prevalence data for LUAD are currently lacking. However, it is important to recognize that lung cancer accounts for over 10% of all new cancer cases and more than 18% of cancer-related deaths worldwide^22^.

Using the TCGA-LUAD database, we conducted a pioneering study on the relationship between LUAD and DRLs. Our research led to the development of a prognostic model based on 27 DRLs, which showed consistent predictive accuracy across various GEO databases. We calculated risk scores for individual patients, revealing significant differences between high-risk and low-risk cohorts, especially regarding the TME, TMB, and responses to immunotherapeutic and chemotherapeutic treatments.

Research has confirmed the involvement of various lncRNAs in cancer initiation and progression, notably LINC00857, which is overexpressed in a range of cancers and regulates multiple cellular activities related to cancer development such as invasion, migration, growth, and apoptosis^24^. A key study showed LINC00857's ability to inhibit cell proliferation across various lung cancer cell lines, highlighting its potential as a therapeutic target^25^. Concurrently, another study identified MIR-210 as a crucial regulator in LUAD, impacting patient survival outcomes through its effects on VEGF expression^26^.

Through detailed KEGG pathway analysis, we found that genes differentially expressed between high-risk and low-risk cohorts were mainly involved in neuroactive ligand-receptor interactions. This is crucial given the hypothesis that GABA receptors play a key role in regulating cell proliferation, a hallmark of cancer^27^. It is imperative to underscore that dysregulated cell proliferation is emblematic of oncogenic processes. Similarly, GO analysis illuminated the role of genes in the negative regulation of peptidase activity, with studies indicating Dipeptidase-2's impact on E-cadherin levels, affecting cell migration, cancer stem cell dynamics, and drug resistance, which in turn affects the survival of LUAD patients^28^.

The interaction between B cells and LUAD has been emphasized, with tumor-infiltrating B cells potentially affecting clinical outcomes in anti-PD-L1 immunotherapy^29^. Dendritic cells, vital for initiating immune responses against tumors, have been identified as diminished in functionality in lung cancer patients, yet remain central to numerous immunotherapeutic strategies^30,31^. In the LUAD tumor microenvironment, the presence of CD8 + T cells, particularly effector memory cells, is associated with improved outcomes^32^. Eosinophils and mast cells also play complex roles in tumor dynamics, with the latter linked to better survival in early-stage LUAD^33,34^. Plasmacytoid dendritic cells have been noted for their positive impact on therapeutic responses and prognosis in LUAD^30,35^.

In our study, we found that LUAD patients in the low-risk group exhibited a significantly enhanced response to immunotherapeutic interventions compared to their high-risk counterparts. We also noted increased expression of various immune checkpoint-associated genes in the low-risk cohort. This elevated expression appears to contribute to the improved immunotherapeutic responsiveness of this group, a conclusion supported by the IPS from the TCIA database. Recent studies have increasingly highlighted the significance of TLS as crucial prognostic markers in oncology, influencing antitumor immune responses, predicting therapeutic outcomes, and correlating with lower recurrence rates^36–38^. In line with this, our data confirm a higher TLS score in the low-risk group. Furthermore, when divided by high and low scores, cohorts with higher TLS scores consistently demonstrated more favorable prognostic outcomes.

## Conclusion

In conclusion, our findings suggest that our prognostic framework has the potential to enhance the accuracy of predicting outcomes for both immunotherapeutic and chemotherapeutic interventions in lung adenocarcinoma patients. This model is poised to provide clinicians with detailed insights into patient responsiveness, thereby aiding in the refinement of therapeutic approaches. Nonetheless, it is crucial to acknowledge the inherent limitations of our study. These include the need for extensive experimental validation and the necessity for broader, multi-institutional studies to strengthen the robustness and universal applicability of the predictive framework.

### Supplementary Information

Supplementary Information.

## Data Availability

The public datasets were obtained from TCGA (https://portal.gdc.cancer.gov/) and GEO (https://www.ncbi.nlm.nih.gov/geo/). GEO Accession Numbers: GSE31210, GSE30219, and GSE50081.

## Abbreviations

lncRNAs: Long non-coding RNAs
LUAD: Lung adenocarcinoma
DRLs: Disulfidptosis-related lncRNAs
TCGA: The Cancer Genome Atlas
C-index: Concordance index
ROC: Receiver operating characteristic
KEGG: Kyoto Encyclopedia of Genes and Genomes
GO: Gene Ontology
KM: Kaplan–Meier
OS: Overall survival
TMB: Tumor mutational burden
DFS: Disease-free survival
SCLC: Small cell lung cancer
NSCLC: Non-small cell lung cancer
ICIs: Immune checkpoint inhibitors
CTLA-4: Cytotoxic T-lymphocyte antigen 4
PD-1: Programmed cell death protein-1
PD-L1: Programmed death-ligand-1
GLUT: Glucose transporter
TME: Tumor microenvironment
TKI: Tyrosine kinase inhibitor
GEO: Gene Expression Omnibus
TPM: Transcripts per million
DRGs: Disulfidptosis-related genes
Lasso: Least Absolute Shrinkage and Selection Operator
RSF: Random Survival Forests
Enet: Elastic Net
SuperPC: Supervised Principal Components
SVM: Survival-Support Vector Machines
GBM: Gradient Boosting Machine
AUC: The area under the ROC curve
DCA: Decision curve analysis
DEGs: Differentially expressed genes
GSEA: Gene Set Enrichment Analysis
ssGSEA: Single sample genome enrichment analysis
TIDE: Tumor Immune Dysfunction and Exclusion
ICB: Immune checkpoint blockade
IPS: Immune Prognostic Score
TCIA: The Cancer Immunome Atlas
TLS: Tumor-infiltrating lymphocytes
ICGs: Immune checkpoint genes
IC50: Half maximal inhibitory concentration
CI: Confidence interval
PCA: Principal component analysis
