# Author Correction: Developing a prognostic model using machine learning for disulfidptosis related lncRNA in lung adenocarcinoma

**DOI:** 10.1038/s41598-024-64894-9

**Published:** 2024-06-14

**Authors:** Yang Pan, Xuanhong Jin, Haoting Xu, Jiandong Hong, Feng Li, Taobo Luo, Jian Zeng

**Affiliations:** 1 Department of Pulmonary Surgery, Zhejiang Cancer Hospital, Hangzhou Institute of Medicine (HIM), Chinese Academy of Sciences, Hangzhou, China; 2 Postgraduate Training Base Alliance of Wenzhou Medical University (Zhejiang Cancer Hospital), Hangzhou, China; 3 Department of Medical Oncology, Sir Run Run Shaw Hospital, College of Medicine, Zhejiang University, Hangzhou, China; 4 School of Medicine, Shaoxing University, Shaoxing, China

Correction to: *Scientific Reports* 10.1038/s41598-024-63949-1, published online 07 June 2024

The original version of this Article contained errors. In the Methods, under the subheading ‘Single-cell analysis and ceRNA network prediction’,

“We then conducted a single-cell analysis of LINC00857 with TISCH (http://tisch.comp-genomics.org/home/) and proceeded with a ceRNA analysis.”

now reads:

“We subsequently performed a single-cell analysis utilizing the TISCH database (http://tisch.comp-genomics.org/home/) and conducted a comprehensive ceRNA analysis.”

In the Results, the subheading ‘Single-cell expression levels of LINC00857’,

now reads:

‘Single-cell expression levels of LINC01003’

The original Article has been corrected.

