# Supplementary material for: Developing a prognostic model using machine learning for disulfidptosis related lncRNA in lung adenocarcinoma
Source: Sci Rep. 2024 Jun 7;14:13113. doi: 10.1038/s41598-024-63949-1 (PMC11161591; doi:10.1038/s41598-024-63949-1)
Supplement: Supplementary file 1 — Supplementary Information. [file 41598_2024_63949_MOESM1_ESM.docx]

# ***Supplementary Table 1*** Detailed data of the 3 GEO datasets.

|  | Number of samples | Number of lung adenocarcinoma samples | Number of samples included in the analysis |
| --- | --- | --- | --- |
| GSE31210 | 226 | 226 | 226 |
| GSE30219 | 307 | 85 | 83 |
| GSE50081 | 181 | 127 | 127 |

# ***Supplementary Table 2*** 27 lncRNAs that formed prognostic model.

| lncRNAs |
| --- |
| ACVR2BAS1 |
| ARIH2OS |
| ATXN1AS1 |
| DLGAP1AS1 |
| DLGAP1AS2 |
| FOXD2AS1 |
| LINC00857 |
| LINC01003 |
| LINC01806 |
| LINC01936 |
| LINC02693 |
| LINC02861 |
| LYRM4AS1 |
| MIR155HG |
| MIR200CHG |
| MIR210HG |
| MIR34AHG |
| NKILA |
| NRAV |
| NUP50DT |
| OGFRP1 |
| PAN3AS1 |
| PCBP1AS1 |
| TMPOAS1 |
| UBE2D3AS1 |
| ZEB1AS1 |
| ZNF710AS1 |

# ***Supplementary Table 3*** Univariate and multivariate Cox analyses of OS in TCGA-LUAD patients.

|  | Univariate | | Multivariate | |
| --- | --- | --- | --- | --- |
| Variables | HR (95% CI) | p Value | HR (95% CI) | p Value |
| Gender | 1.09(0.81-1.46) | 0.579 | 0.85(0.63-1.16) | 0.318 |
| Age | 1.01(0.99-1.02) | 0.409 | 1.01(1.00-1.03) | 0.093 |
| Stage | 1.64(1.43-1.89) | <0.001 | 1.46(1.16-1.84) | 0.001 |
| Risk Score | 2.72(2.25-3.29) | <0.001 | 2.49(2.04-3.04) | <0.001 |
| T | 1.47(1.23-1.77) | <0.001 | 1.03(0.85-1.24) | 0.796 |
| N | 1.61(1.36-1.91) | <0.001 | 1.05(0.83-1.33) | 0.686 |
| M | 1.47(1.09-1.99) | 0.012 | 0.92(0.65-1.30) | 0.642 |

# ***Supplementary Table 4*** Multicollinearity diagnosis based on multivariate Cox analyses.

| Variables | VIF |
| --- | --- |
| Gender | 1.04 |
| Age | 1.01 |
| Stage | 2.41 |
| Risk score | 1.11 |
| T | 1.22 |
| N | 1.94 |
| M | 1.23 |

***Supplementary Figure S1*** (A-C) Differences in disease-free survival (DFS) between the two risk groups. (D) C-index of the risk model.

***Supplementary Figure S2*** Differences in risk score for (A)stage I, (B)stage II, and (C)stage Ⅲ/Ⅳ lung adenocarcinoma. (D)Differences in risk scores in patients with genetic mutations. Differences in risk scores between smoking and non-smoking groups:(E) GSE31210, (F)GSE50081. (G) The distribution of DEGs between the low-risk and high-risk cohorts.

***Supplementary Figure S3*** High-risk and low-risk group differences in (A)ESTIMATE score, (B)tumor purity. (C)3D PCA plot for samples using 2500 genes having largest variance.

***Supplementary Figure S4*** (A-B) Single-cell analysis of LINC01003. (C) The ceRNA network of LINC00857.


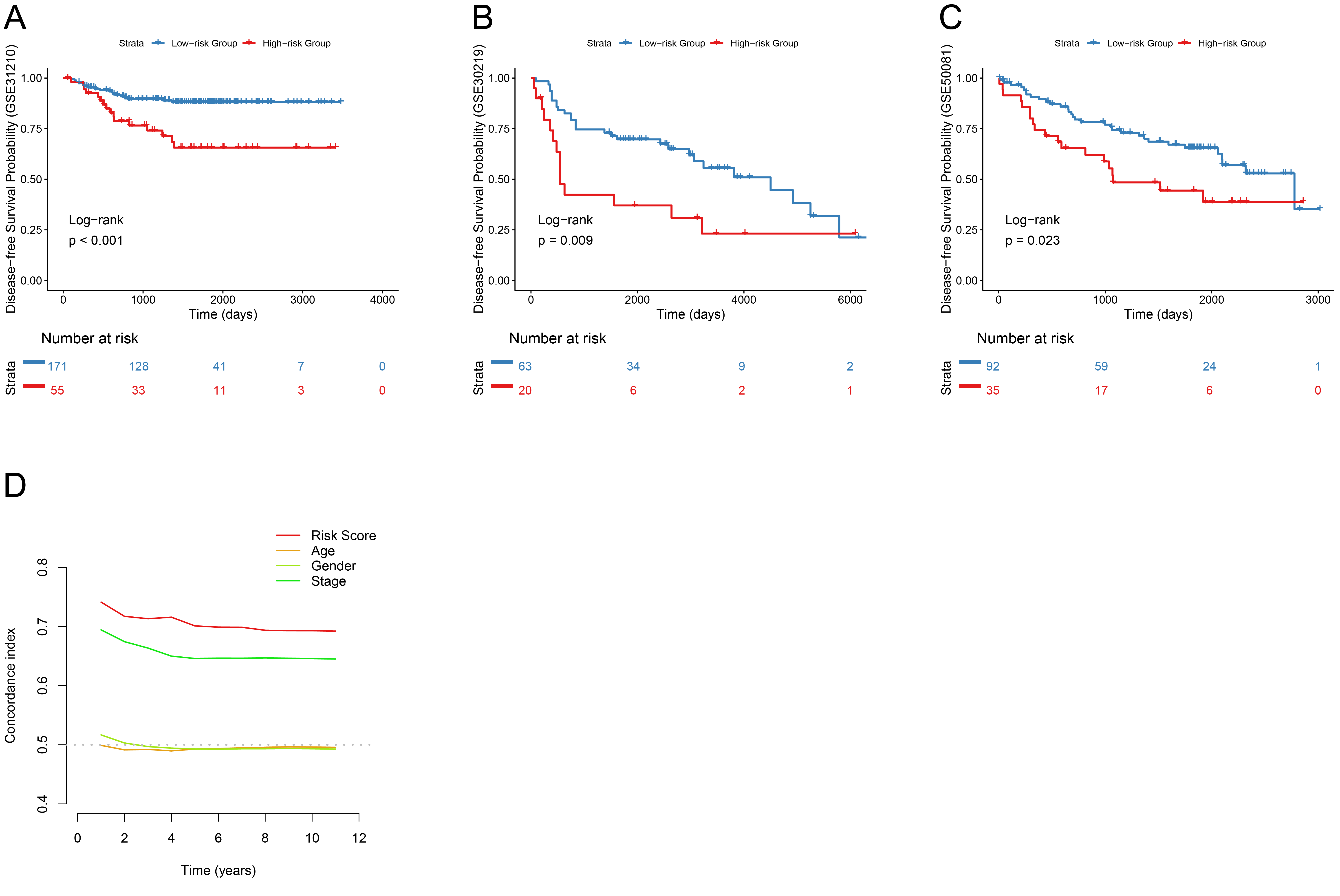


# ***Supplementary Figure S1*** (A-C) Differences in disease-free survival (DFS) between the two risk groups. (D) C-index of the risk model.


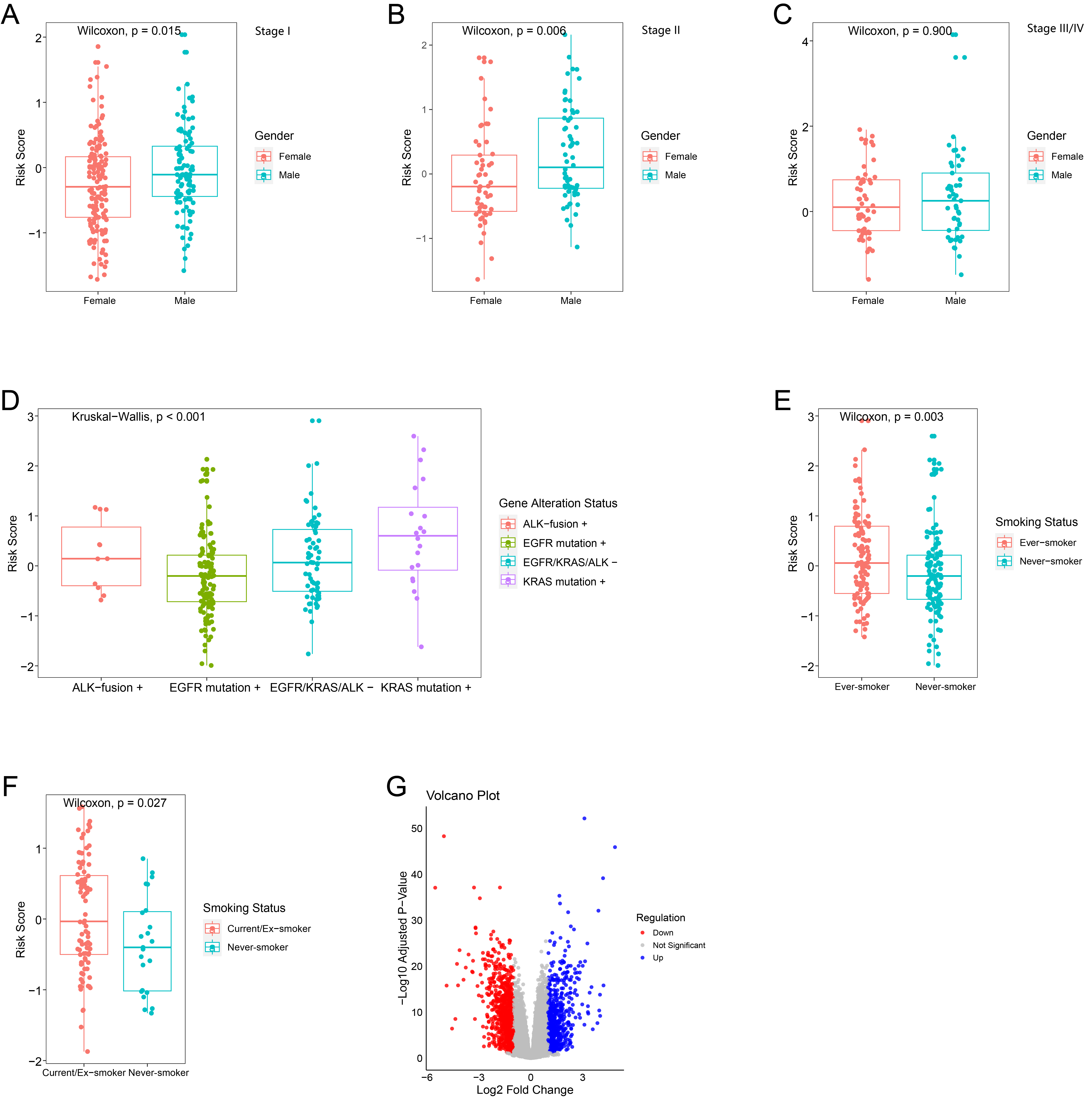


# ***Supplementary Figure S2*** Differences in risk score for (A)stage I, (B)stage II, and (C)stage Ⅲ/Ⅳ lung adenocarcinoma. (D)Differences in risk scores in patients with genetic mutations. Differences in risk scores between smoking and non-smoking groups:(E) GSE31210, (F)GSE50081. (G) The distribution of DEGs between the low-risk and high-risk cohorts.


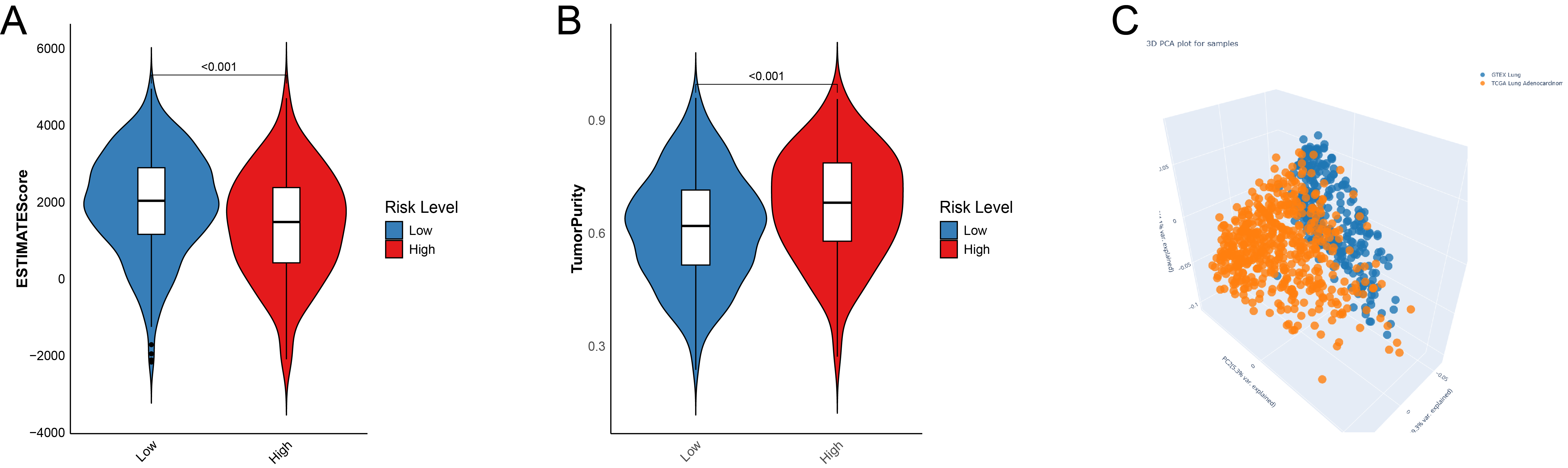


# ***Supplementary Figure S3*** High-risk and low-risk group differences in (A)ESTIMATE score, (B)tumor purity. (C)3D PCA plot for samples using 2500 genes having largest variance.


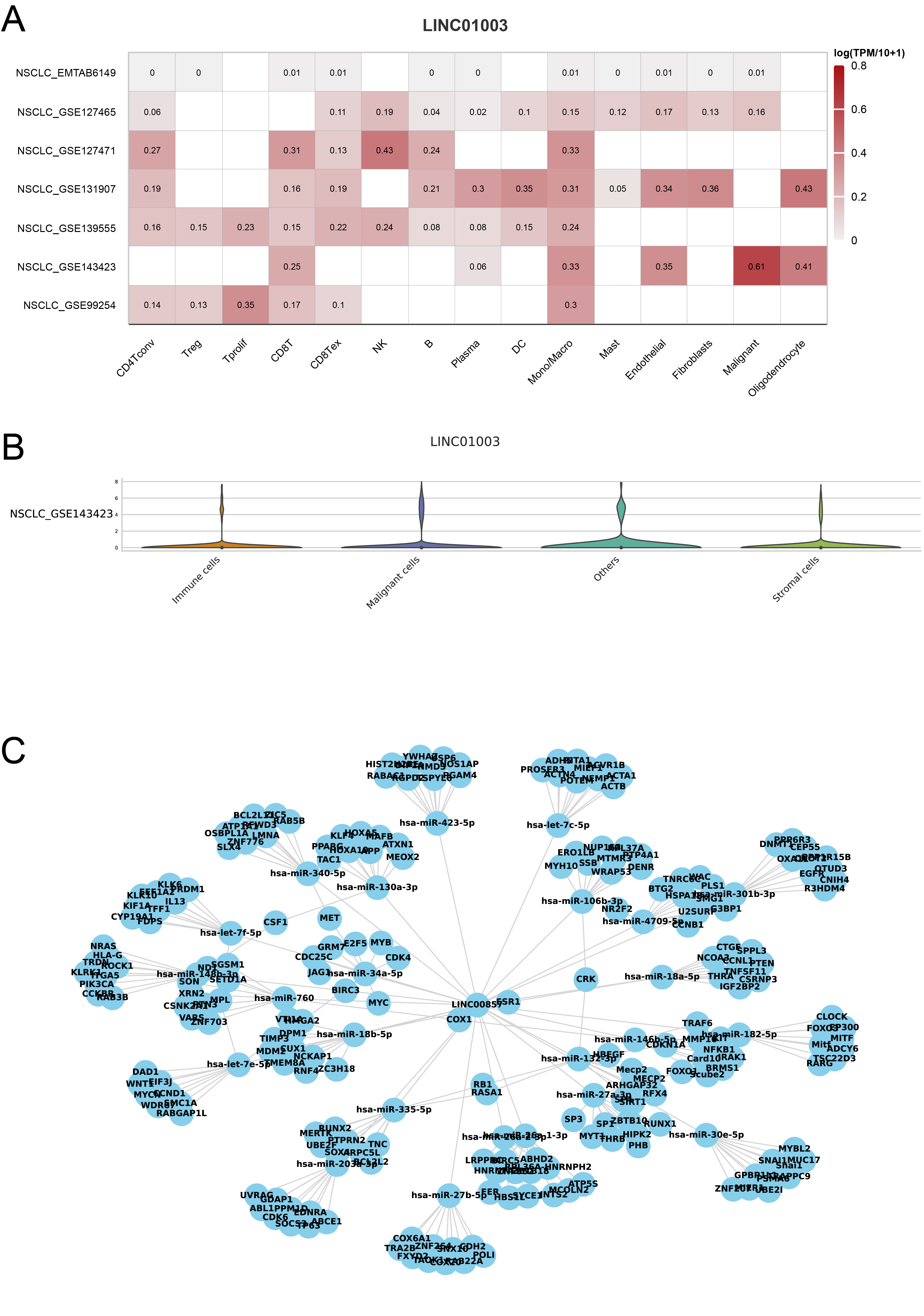


# ***Supplementary Figure S4*** (A-B) Single-cell analysis of LINC01003. (C) The CeRNA network of LINC00857.
